# Supplementary material for: Sexually Dimorphic Effects of Histamine Degradation by Enteric Glial Histamine N-Methyltransferase (HNMT) on Visceral Hypersensitivity
Source: Biomolecules. 2023 Nov 14;13(11):1651. doi: 10.3390/biom13111651 (PMC10669271; doi:10.3390/biom13111651)

Figure 2 B

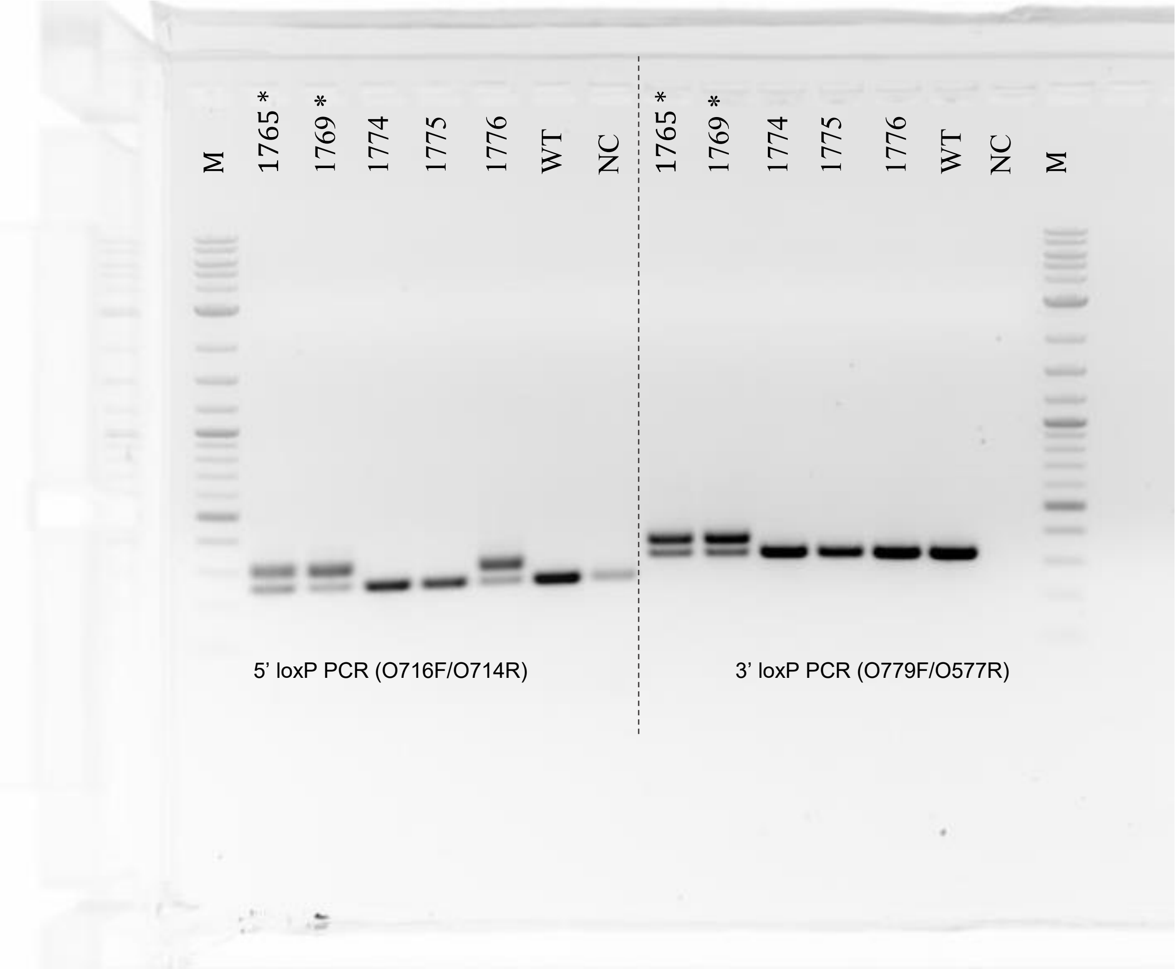

M = Ladder molecular weight; WT = Wild-type; NC = Negative Control

Figure_S1 E

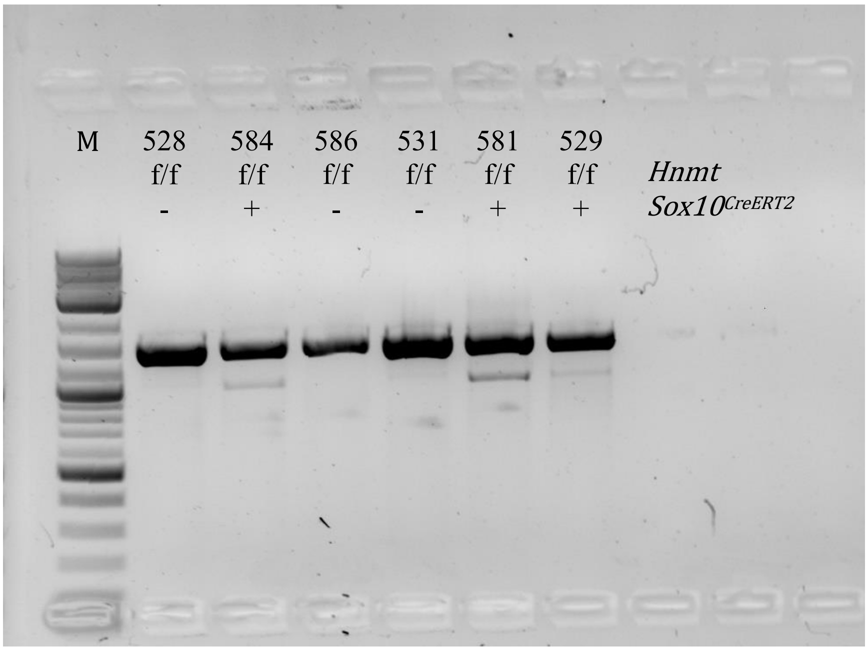

Figure S2 A

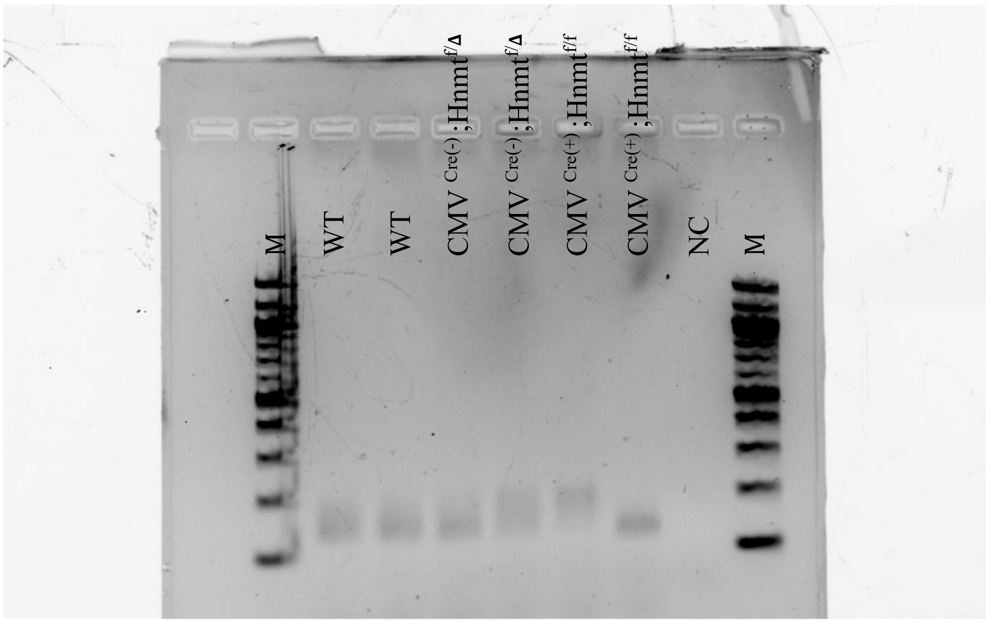

Figure S2 B

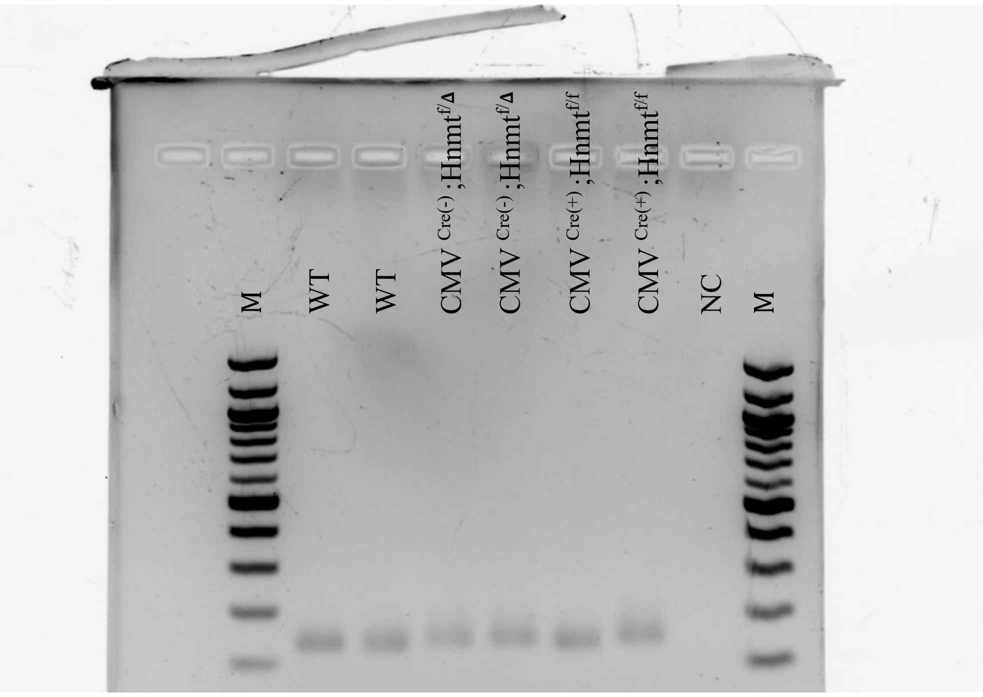

Figure S2 C

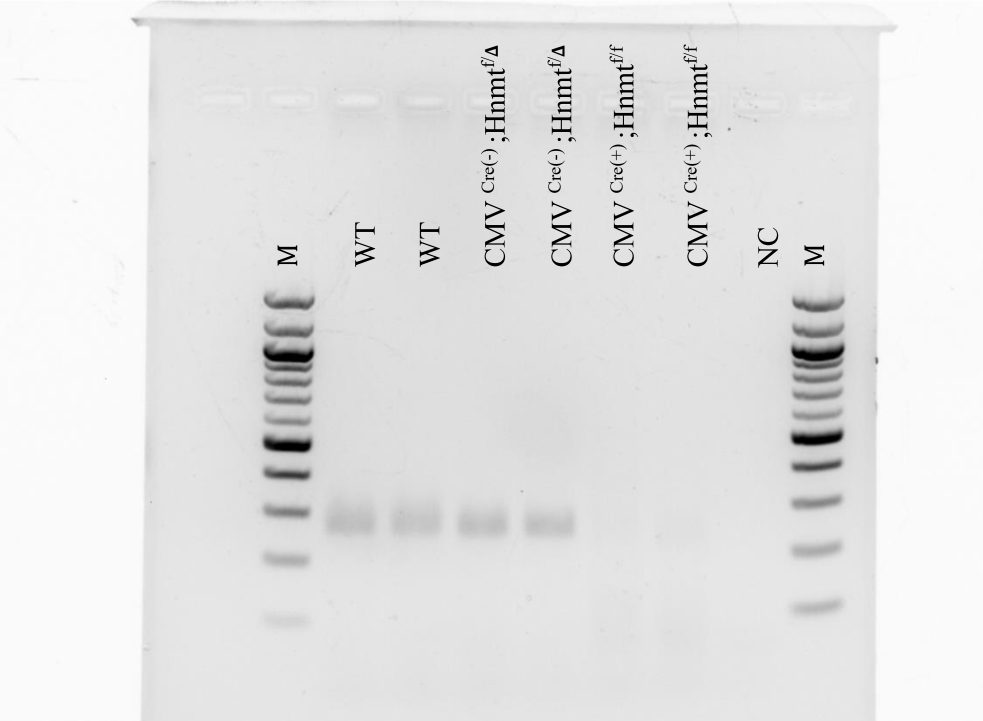

Figure S2 D

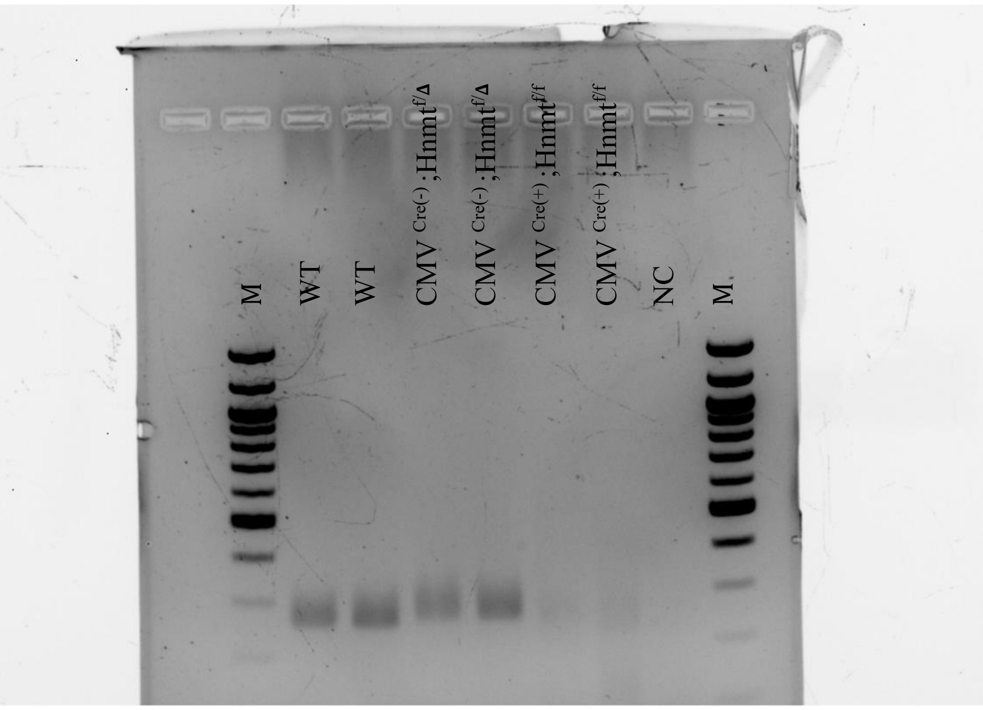

Figure S2 E

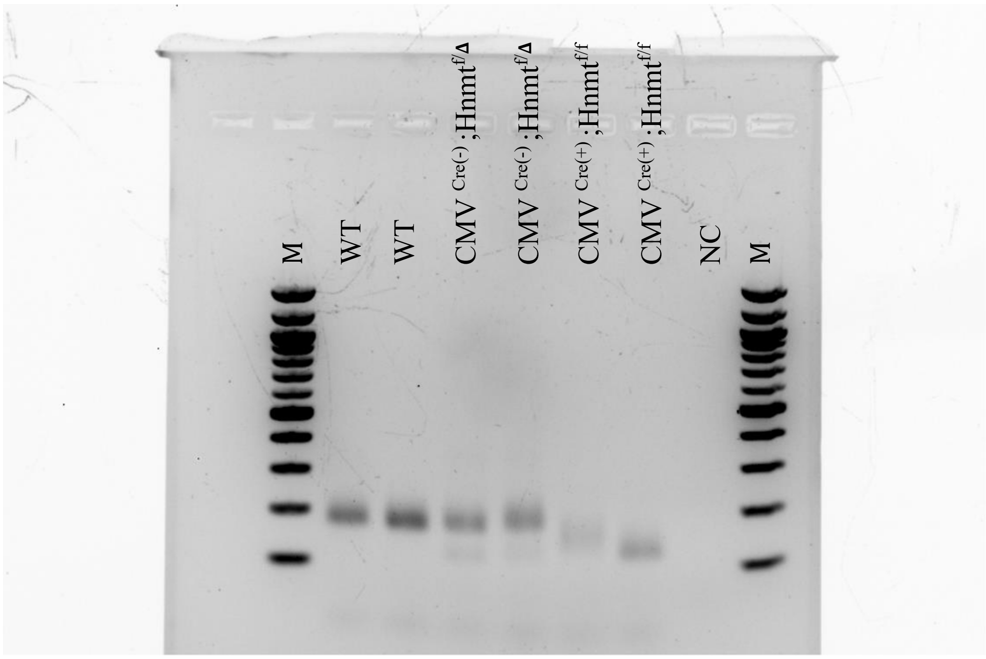

Figure_S2 F

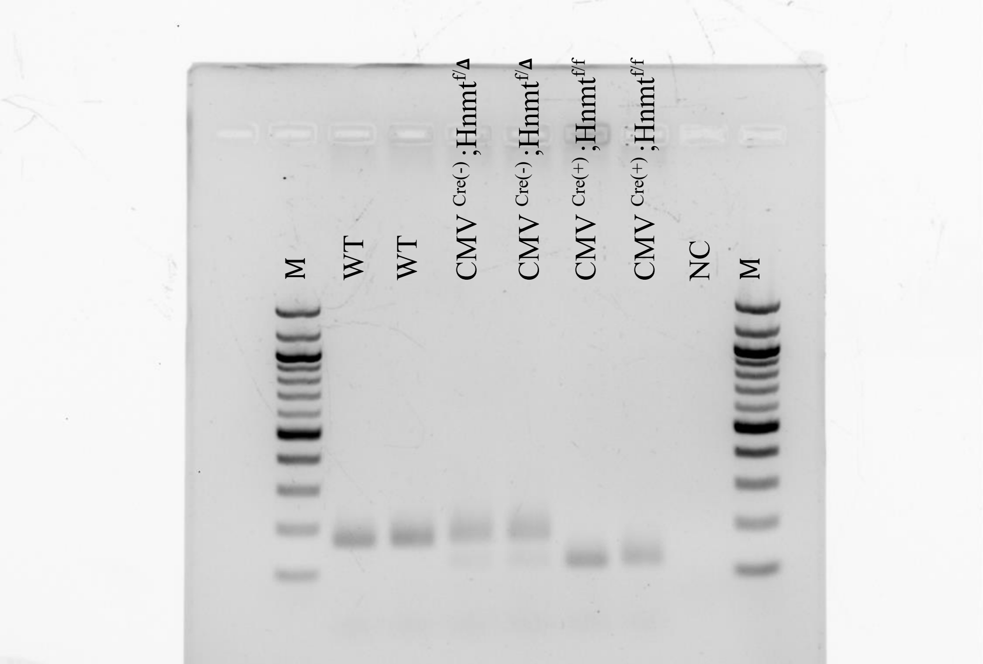

Supplement: Supplementary file 1 [file biomolecules-13-01651-s001.zip › Glia_HNMT_Original Gel Images.pdf]
