# Supplementary material for: Sexually Dimorphic Effects of Histamine Degradation by Enteric Glial Histamine N-Methyltransferase (HNMT) on Visceral Hypersensitivity
Source: Biomolecules. 2023 Nov 14;13(11):1651. doi: 10.3390/biom13111651 (PMC10669271; doi:10.3390/biom13111651)
Supplement: Supplementary file 1 [file biomolecules-13-01651-s001.zip › Glia_HNMT_Supplemental.pdf]

Figure S1. Targeting of the mouse histamine N-methyltransferase (*Hnmt*) locus. (**A**) Schematic of the targeted mouse *Hnmt* genomic locus with coding exons in blue, untranslated regions in light blue, and loxP sites denoted by black arrowheads. (**B**) Location and exact sequences of gRNA target regions within intron 2 and intron 3; dotted red line denotes DSB, PAM is highlighted and sequences corresponding to gRNA protospacer are underlined. (**C**) Sanger sequencing chromatograms of targeted regions after PCR cloning from G1 offspring gDNA, highlighted regions mark LoxP sequences. (**D**) Sanger sequencing of PCR amplicons spanning the 401 bp deletion region following Cre/LoxP recombination from a sample of $CMV^{Cre(+)}$;$Hnmt^{f/f}$ animal. Dotted line denotes deletion breakpoint. Shaded region is the single LoxP site remaining after recombination. (**E**) Deletion of the *Hnmt* floxed region driven by $Sox10^{CreERT2}$ in intestinal tissue. Genomic DNA (gDNA) was extracted from Ileum rod samples from $Sox10^{CreERT2}$;*Hnmt* mice. Note a lower molecular weight band is present in samples positive for $Sox10^{CreERT2}$. Abbreviations: DSB - double strand DNA break, gRNA - guide RNA, PAM - protospacer adjacent motif.

Figure S2. Effects of enteric glial histamine N-methyltransferase (*Hnmt*) relative expression in mouse colon. (**A** and **B**) Representative qPCR gels utilizing *Hnmt* exon 1 primers from colon samples in WT and CMV^{Cre};*Hnmt* mice in males (**A**) and females (**B**). (**C** and **D**) Representative qPCR gels utilizing *Hnmt* exon 3 primers from colon samples in WT and CMV^{Cre};*Hnmt* mice in males (**C**) and females (**D**). (**E** and **F**) Representative qPCR gels utilizing *Hnmt* exon 2-4 primers from colon samples in WT and CMV^{Cre};*Hnmt* mice in males (**E**) and females (**F**). (**G**) Quantification of qPCR data of relative expression

of *Hnmt* exon 2-4 normalized to *Rps6* in WT, $CMV^{Cre(-)}$*;*$Hnmt^{f/\Delta}$, and $CMV^{Cre(+)}$*;*$Hnmt^{f/f}$ mice. (**H** and **I**) Quantification of qPCR data of relative expression of *Hnmt* exon 3 and exon 2-4 to *Hnmt* exon 1 in WT, $CMV^{Cre(-)}$*;*$Hnmt^{f/\Delta}$, and $CMV^{Cre(+)}$*;*$Hnmt^{f/f}$ mice (**H** and **I**, respectively). n = 5-9 animals per group, **P < 0.01, ***P < 0.001, One-way ANOVA.

Figure S3. Effects of glial histamine N-methyltransferase (*Hnmt*) ablation on Diamine oxidase (*Dao*) and N-acetyltransferase 2 (*Nat2*) relative expression in mouse ileum of $Sox10^{CreERT2}$*;Hnmt* and WT littermates. (**A** and **B**) Quantification of qPCR data of relative expression of *Dao* and *Nat2* normalized to *Rps6* (**A** and **B**, respectively) in males and females from WT littermates and $Sox10^{CreERT2}$*;Hnmt*. n = 6 animals per group, 2-way ANOVA.

Figure S1.

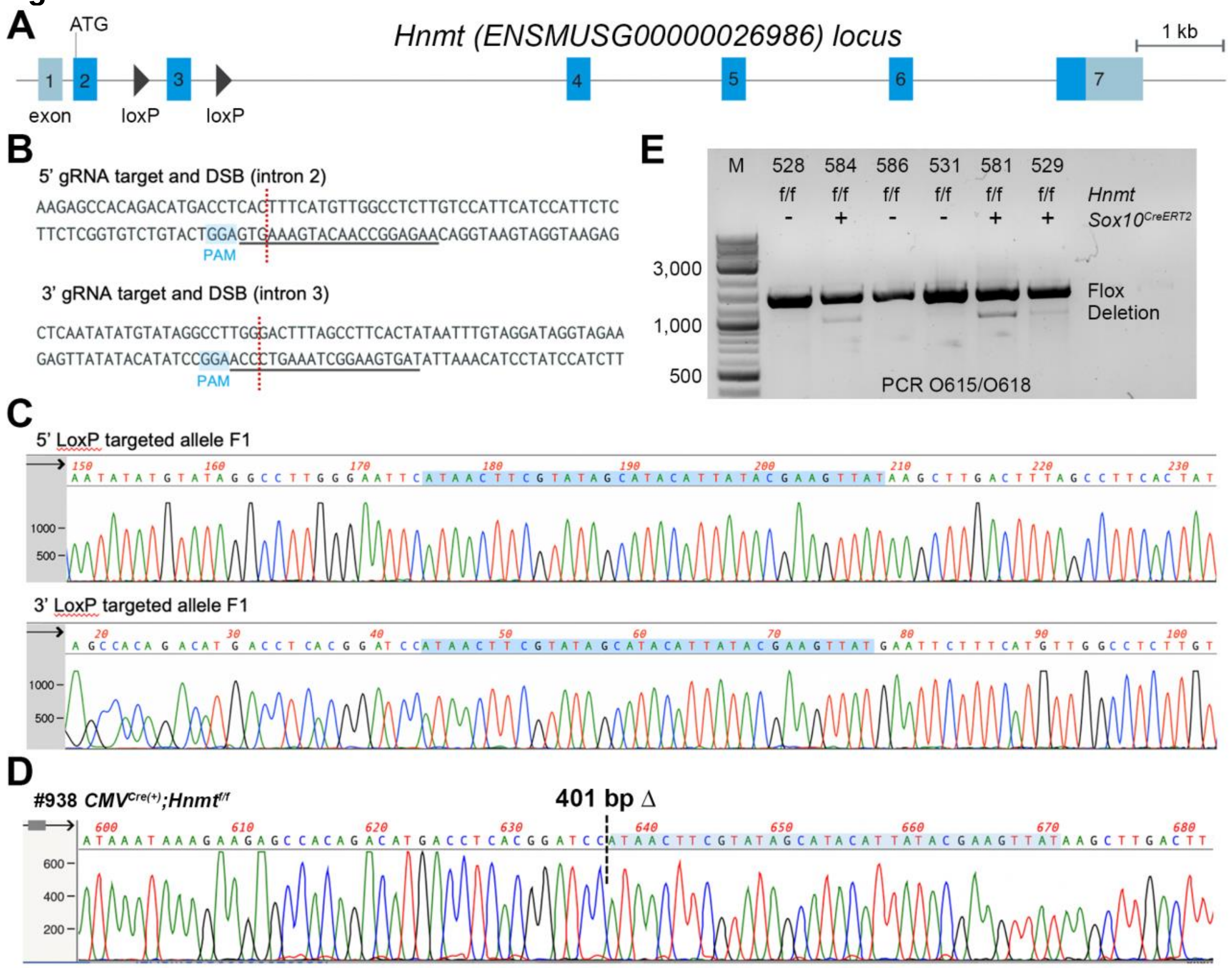
A
ATG
Hnmt (ENSMUSG00000026986) locus
1 kb
exon
loxP
loxP
B
5' gRNA target and DSB (intron 2)
AAGAGCCACAGACATGACCTCACTTTCATGTTGGCCTCTTGTCCATTCATCCATTCTC
TTCTCGGTGTCTGTACTGGAGTGAAAGTACAACCGGAGAACAGGTAAGTAGGTAAGAG
PAM
3' gRNA target and DSB (intron 3)
CTCAATATATGTATAGGCCTTGGGACTTTAGCCTTCACTATAATTTGTAGGATAGGTAGAA
GAGTTATATACATATCCGGAACCCTGAAATCGGAAGTGATATTAAACATCCTATCCATCTT
PAM
C
5' LoxP targeted allele F1
3' LoxP targeted allele F1
D
#938 CMVCre(+);Hnmtf/f
401 bp Δ
E
M 528 584 586 531 581 529
f/f f/f f/f f/f f/f f/f Hnmt
- + - - + + Sox10CreERT2
3,000
1,000
500
Flox
Deletion
PCR O615/O618

Figure S2.

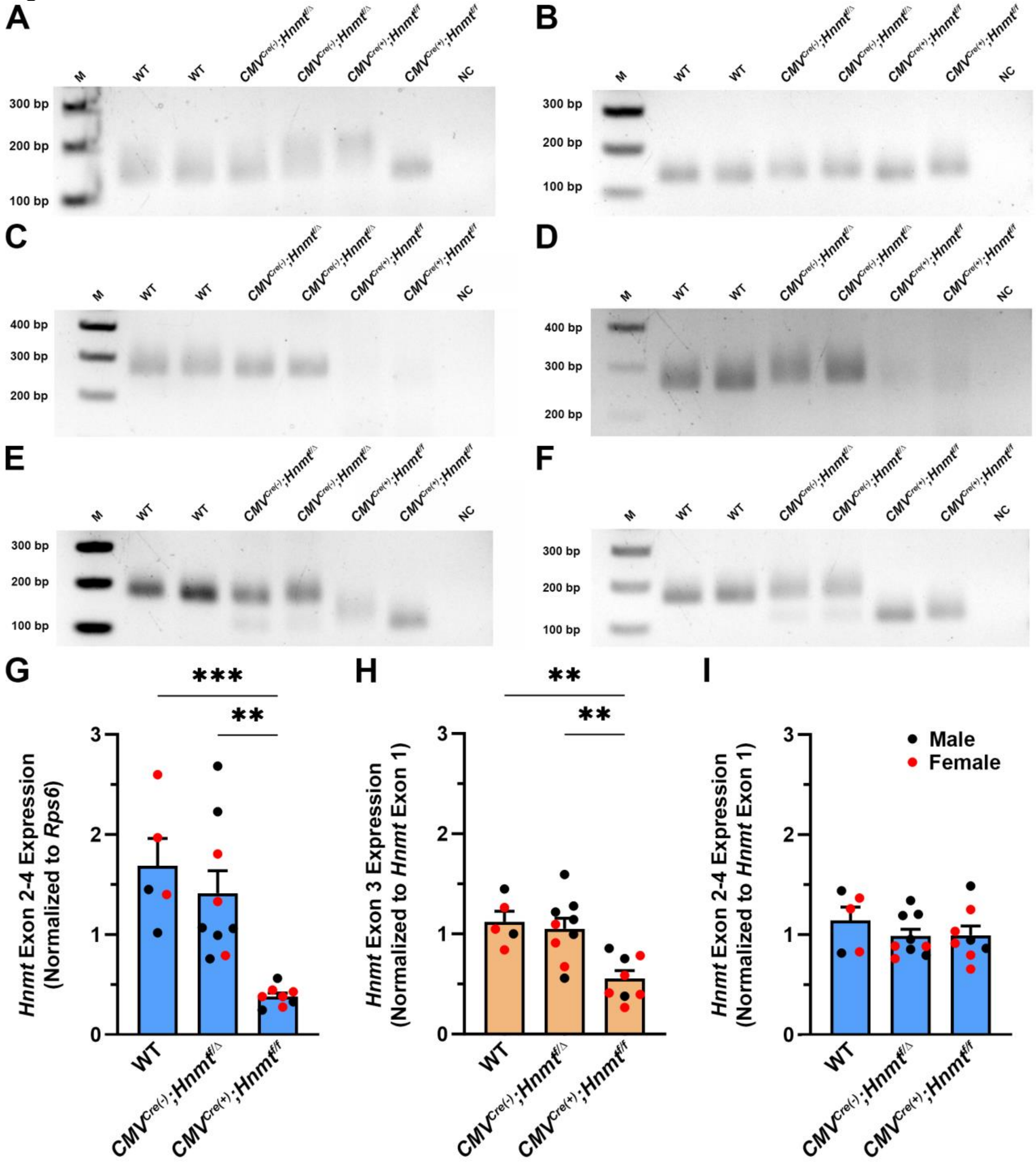
A
B
C
D
E
F
G
H
I
M
WT
$CMV^{Cre(-)}$;$Hnmt^{f/\Delta}$
$CMV^{Cre(+)}$;$Hnmt^{f/f}$
NC
100 bp
200 bp
300 bp
400 bp
***
**
Hnmt Exon 2-4 Expression (Normalized to *Rps6*)
Hnmt Exon 3 Expression (Normalized to *Hnmt* Exon 1)
Hnmt Exon 2-4 Expression (Normalized to *Hnmt* Exon 1)
Male
Female

Figure S3.

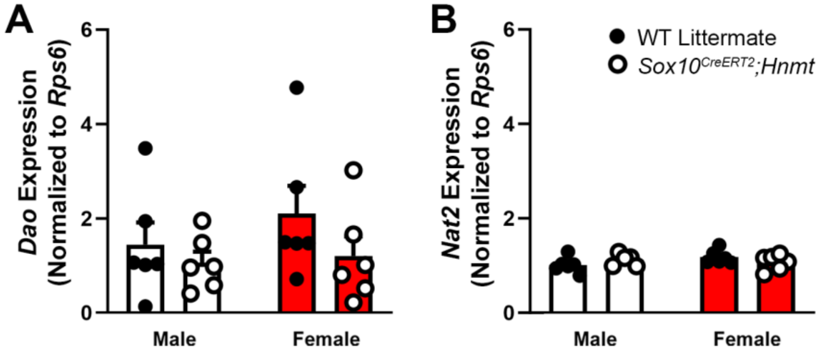

A
B
Dao Expression
(Normalized to *Rps6*)
Nat2 Expression
(Normalized to *Rps6*)
6
4
2
0
Male
Female
WT Littermate
Sox10[CreERT2]*;Hnmt*
