# Supplementary figures and images for: Sexually Dimorphic Effects of Histamine Degradation by Enteric Glial Histamine N-Methyltransferase (HNMT) on Visceral Hypersensitivity
Source: Biomolecules. 2023 Nov 14;13(11):1651. doi: 10.3390/biom13111651 (PMC10669271; doi:10.3390/biom13111651)

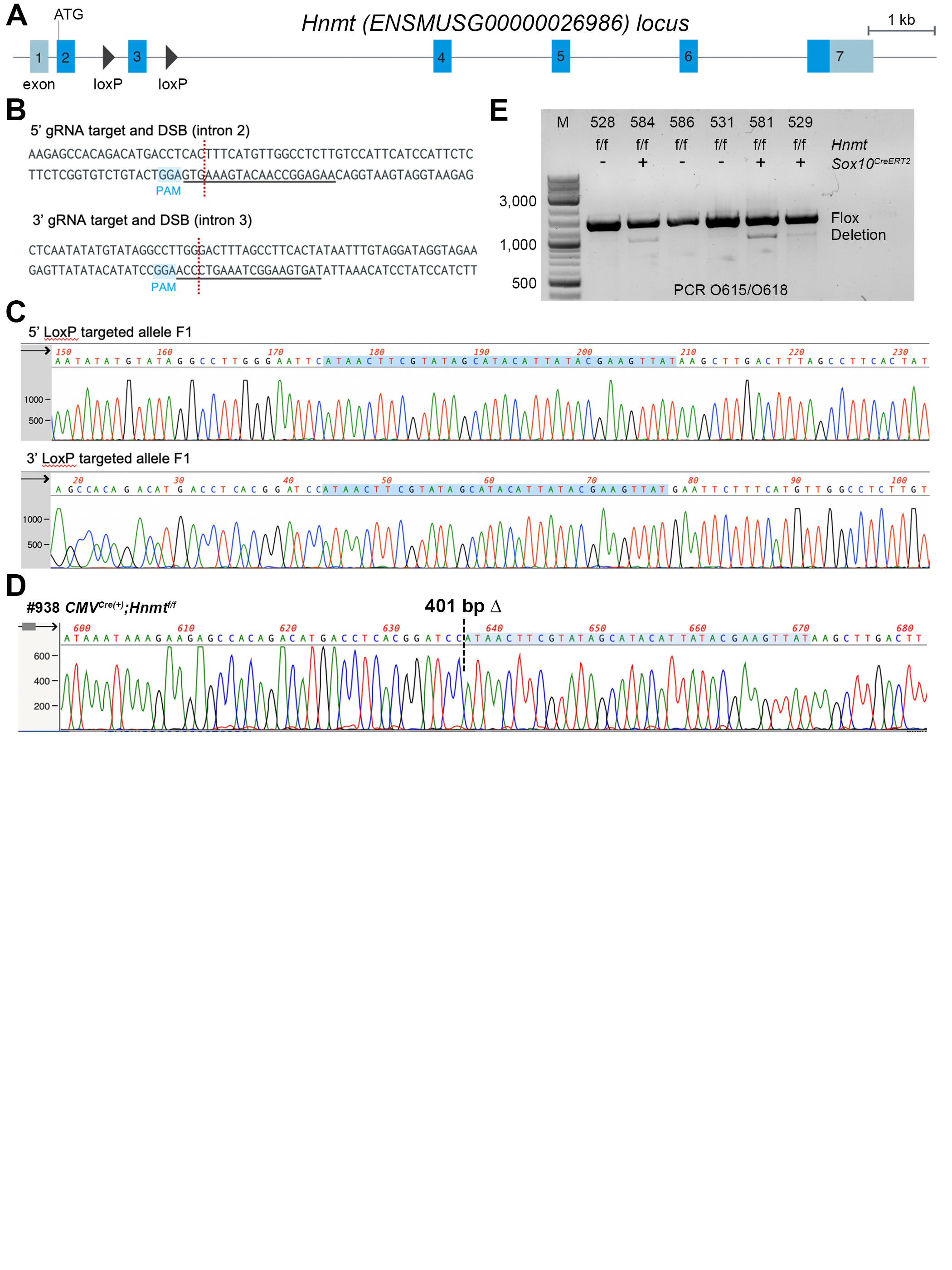

Supplement: Supplementary file 1 [file biomolecules-13-01651-s001.zip › Glia_HNMT_Supplemental_Figures/Supplemental_Figure_1.tif]

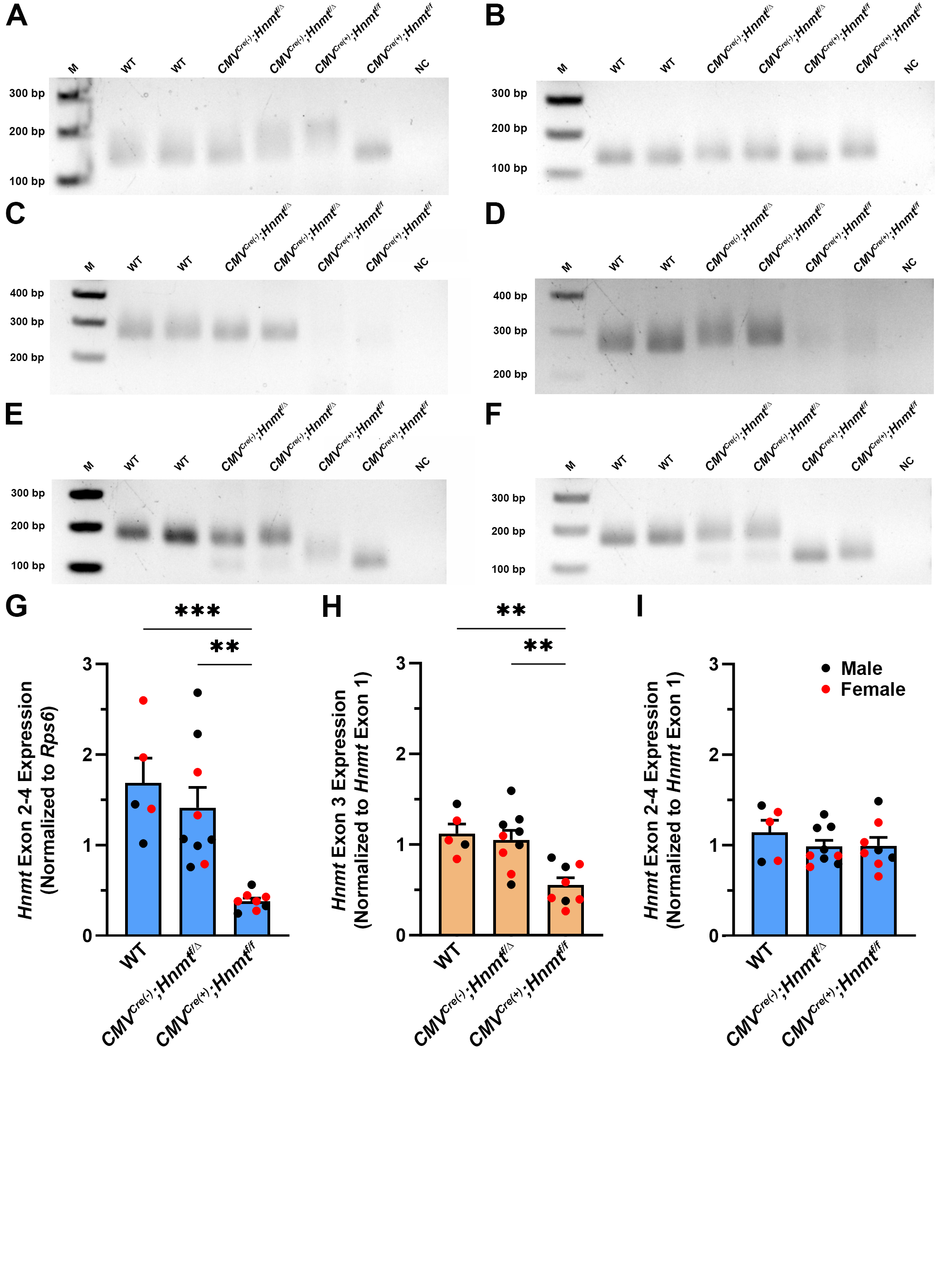

Supplement: Supplementary file 1 [file biomolecules-13-01651-s001.zip › Glia_HNMT_Supplemental_Figures/Supplemental_Figure_2.tif]

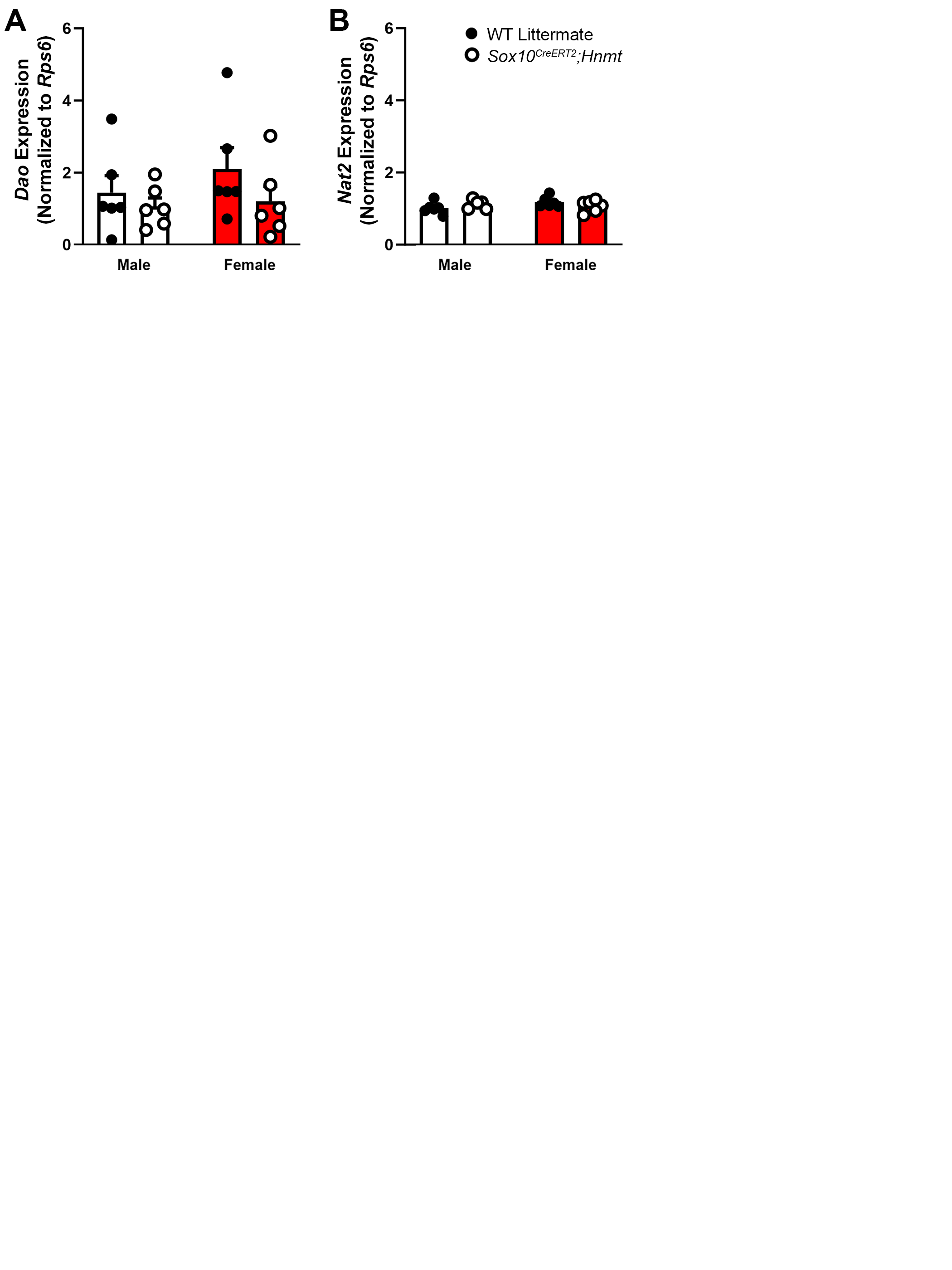

Supplement: Supplementary file 1 [file biomolecules-13-01651-s001.zip › Glia_HNMT_Supplemental_Figures/Supplemental_Figure_3.tif]
